# Acceptance commitment therapy for preschool children : A pilot study

**DOI:** 10.1192/j.eurpsy.2021.1324

**Published:** 2021-08-13

**Authors:** T. Brahim, S. Bouzgarrou, A. Guedria

**Affiliations:** Child And Adolescent Psychiatry Department, Fattouma Bourguiba University Hospital, Monastir, Tunisia, Monastir, Tunisia

**Keywords:** preschool children, regulation, emotion, acceptance therapy

## Abstract

**Introduction:**

Emotion regulation is a key world to understand many human behaviors. Preschool children can exhibite disturbing behaviors that could interfeer with their integration, development and learning abilities. One way to understand these behaviors is their “immature” emotional regulation process. Helping children acquiring this ability can help to solve these disturbances’, which can lead to mental health problems.

**Objectives:**

To experiment with a new acceptance and committment therapy protocol in preschool children

**Methods:**

It is a qualitative interventional study that relies on issues to enable the recognition and regulation of emotions. a protocol was implemented which consists of activities and techniques useful to help children recognize, regulate and accept their emotions, with the support of the educator.

**Results:**

All six selected children accepted to undergo the therapy till the end of the eight sessions. at fisrt, they were enable to recognise some emtions. it was the most difficult for them to recognise body expression of emotion. they had also difficulties find the different worlds to express them and to enact them.Throw different activities, we tried to help children visualize their emotions, imagine them under different faces and play with them. Children were introduced to mindfulness and emotional regulation technics. After One month of the intervention, children were still able to recognize emotions and to propose technics to regulate them. their score on the CBCL were below 70 one month after the intervention.

**Conclusions:**

Even at an early age of 4 years, ACT seems to be possible and helpful for children.

